# Impact of *Helicobacter pylori*-Related Metabolic Syndrome Parameters on Arterial Hypertension

**DOI:** 10.3390/microorganisms9112351

**Published:** 2021-11-14

**Authors:** Jannis Kountouras, Apostolis Papaefthymiou, Stergios A. Polyzos, Georgia Deretzi, Elisabeth Vardaka, Elpidoforos S. Soteriades, Maria Tzitiridou-Chatzopoulou, Paraskevas Gkolfakis, Kyriaki Karafyllidou, Michael Doulberis

**Affiliations:** 1 Second Medical Clinic, School of Medicine, Ippokration Hospital, Aristotle University of Thessaloniki, 54642 Thessaloniki, Greece; appapaef@hotmail.com (A.P.); mariatzitiridou@gmail.com (M.T.-C.); doulberis@gmail.com (M.D.); 2 Department of Gastroenterology, University Hospital of Larisa, 41110 Larisa, Greece; 3 First Laboratory of Pharmacology, School of Medicine, Aristotle University of Thessaloniki, 54124 Thessaloniki, Greece; spolyzos@auth.gr; 4 Multiple Sclerosis Unit, Department of Neurology, Papageorgiou General Hospital, 56403 Thessaloniki, Greece; gderetzi@gmail.com; 5 Department of Nutritional Sciences and Dietetics, School of Health Sciences, International Hellenic University, 57400 Thessaloniki, Greece; evardaka@aqua.theite.gr; 6 Healthcare Management Program, School of Economics and Management, Open University of Cyprus, Nicosia 2252, Cyprus; elpidoforos.soteriades@ouc.ac.cy; 7 Department of Environmental Health, Environmental and Occupational Medicine and Epidemiology (EOME), Harvard T.H. Chan School of Public Health, Boston, MA 02115, USA; 8 School of Healthcare Sciences, Midwifery Department, University of West Macedonia, Koila, 50100 Kozani, Greece; 9 Department of Gastroenterology, Hepatopancreatology and Digestive Oncology, Erasme University Hospital, 1070 Brussels, Belgium; pgkolfakis@med.uoa.gr; 10 Department of Medical Oncology, Institut Jules Bordet, 1000 Brussels, Belgium; 11 Department of Pediatrics, University Children’s Hospital of Zurich, 8032 Zurich, Switzerland; kikh.karafyllidou@gmail.com; 12 Division of Gastroenterology and Hepatology, Medical University Department, Kantonsspital Aarau, 5001 Aarau, Switzerland

**Keywords:** *Helicobacter pylori*, metabolic syndrome, atherosclerosis, non-alcoholic fatty liver disease, arterial hypertension, diet, inflammation

## Abstract

Arterial hypertension is a risk factor for several pathologies, mainly including cardio-cerebrovascular diseases, which rank as leading causes of morbidity and mortality worldwide. Arterial hypertension also constitutes a fundamental component of the metabolic syndrome. *Helicobacter pylori* infection is one of the most common types of chronic infection globally and displays a plethora of both gastric and extragastric effects. Among other entities, *Helicobacter pylori* has been implicated in the pathogenesis of the metabolic syndrome. Within this review, we illustrate the current state-of-the-art evidence, which may link several components of the *Helicobacter pylori*-related metabolic syndrome, including non-alcoholic fatty liver disease and arterial hypertension. In particular, current knowledge of how *Helicobacter pylori* exerts its virulence through dietary, inflammatory and metabolic pathways will be discussed. Although there is still no causative link between these entities, the emerging evidence from both basic and clinical research supports the proposal that several components of the *Helicobacter pylori* infection-related metabolic syndrome present an important risk factor in the development of arterial hypertension. The triad of *Helicobacter pylori* infection, the metabolic syndrome, and hypertension represents a crucial worldwide health problem on a pandemic scale with high morbidity and mortality, like COVID-19, thereby requiring awareness and appropriate management on a global scale.

## 1. Introduction

Arterial hypertension is one of the most important components of the metabolic syndrome (MetS) and represents a global major health burden with implications for individual and public health, as well as direct and indirect healthcare costs. It is a substantial factor for the development of atherosclerosis, with a high global incidence and prevalence that continues to increase and contributes to worldwide morbidity and mortality [1]. It constitutes a major risk factor for MetS-related cardiovascular disease and the leading cause of mortality related with MetS-associated chronic kidney failure, ischemic heart disease and stroke [2,3]. In 2015, a total of 1.13 billion adults had arterial hypertension worldwide [4]; this figure is predicted to increase to 1.56 billion by 2025 [5]. Notably, an estimated 7.7–10.4 million annual deaths are attributable to arterial hypertension [6,7].

Although the pathogenesis of arterial hypertension is not entirely understood, there is support for the idea that its pathogenesis predominantly consists of a noxious interplay among vascular, renal, neural, and hormonal mechanisms, of which increased activation of the sympathetic nervous system (SNS) and the renin–angiotensin–aldosterone system (RAAS) prevail [8]; RAAS dysregulation, including the systemic and brain RAAS, has been recognized as one of the main causes of several types of arterial hypertension [9]. RAAS overactivation also contributes to MetS-related obesity and cardiovascular morbidity and mortality [10,11].

Specifically, potential causative agents of hypertension include the following (Table 1): (1) genetic factors [12]; (2) diet (particularly sodium and potassium intake, as well as western diet) [13,14,15]; (3) adiposity [16]; (4) hyperinsulinemia and insulin resistance (IR) [17]; (5) smoking [18]; (6) endothelial dysfunction linked with MetS [19], with excessive release of vasoconstrictive agents and defective secretion of smooth-muscle relaxing mediators, such as nitric oxide [20]; (7) gut microbiota dysbiosis connected with MetS [15,21,22,23]; (8) inflammatory mechanisms, including pro-inflammatory cytokines and chemokines overexpression, cell infiltration and oxidative stress—all induced by excessive immune system stimulation—that are strongly upregulated in the hypertensive setting [24,25]; (9) innate and adaptive immune system involvement [25,26]; (10) nonalcoholic fatty liver disease (NAFLD), also closely associated with MetS [27]; (11) MetS-related brain neurodegenerative disorders, through disruption to the blood–brain barrier, triggering neuroinflammation, and exacerbation of amyloid disorders and decreased function of the cerebral blood vessels, including reduced cerebral blood flow, altered brain autoregulation, and compromised neurovascular coupling [28,29,30,31]; (12) MetS–related cancer development [32,33].

Likewise, *Helicobacter pylori* (*H. pylori*) infection is a global public health problem [34], with higher burdens in the developing nations [34,35], mostly owing to low socio-economic levels, and the potential drinking of contaminated water. *H. pylori* infection is very common with a mean global prevalence of 58%, partly due to immigrants coming from countries with a high prevalence of *H. pylori* infection [36]. A rather recent global systematic review on the prevalence of *H. pylori* estimated that approximately 4.4 billion individuals were infected from *H. pylori* in 2015 [34]. Several issues, including age, gender, number of family members and financial status, education, lifestyle, health condition, and area of residence could affect the prevalence of *H. pylori* in different populations [34].

Beyond *H. pylori*-related gastric pathologies [37], *H. pylori* infection is also associated with MetS-related systemic pathologies, especially cardio-cerebrovascular, and neurodegenerative diseases, the endpoints of MetS [38,39,40,41,42,43]. It is important to note that almost all the aforementioned potential causative agents involved in the pathogenesis of arterial hypertension are also involved in the pathophysiology of *H. pylori*-related pathologies [36,38,39,40,42,43,44,45,46,47,48,49,50,51,52,53,54,55].

In this study, we aimed to investigate the potential impact of *H. pylori*-related MetS parameters on arterial hypertension, since the triad of *H. pylori* infection, MetS, and hypertension currently represents a fundamental worldwide health problem on a pandemic scale with high morbidity and mortality, like COVID-19, thereby requiring awareness and appropriate management on a global scale [56,57].

## 2. *H. pylori*-Related Arterial Hypertension

Generally, pathogen infection could be one of the inflammatory triggering factors that is connected with the incidence and development of arterial hypertension, including abnormal systolic and/or diastolic pressure [58,59]. Chronic stimulation of inflammatory reactions owing to bacterial infection in the gastrointestinal tract generates induction of MetS-related dyslipidemia, triggers release of C-reactive protein (CRP), escalates blood leukocytes and MetS-related homocysteine, increases the concentrations of MetS-related fibrinogen, induces hypercoagulability, stimulates immune cross-reactivity, and rises pro-inflammatory cytokines and other cytotoxic agents. This intense rise in the release of diverse pro-inflammatory and inflammatory mediators disturbs blood vessel motility and induces endothelial dysfunction, which results in the obstruction of arteries, thereby leading to arterial hypertension and coronary artery disease. In this regard, chronic *H. pylori* infection leads to disturbed immune reactions, which ultimately contribute to arterial hypertension and cardiovascular abnormalities, including coronary artery disease. Specifically, relative data indicate that *H. pylori* is positively correlated with the risk of arterial hypertension [60,61,62]. As a consequence, *H. pylori*-positive hypertensive patients display significantly higher arterial blood pressure than that of hypertensive patients without the infection [62]. Moreover, *H. pylori* eradication has been reported to improve arterial hypertension [63,64].

**Figure 1:**
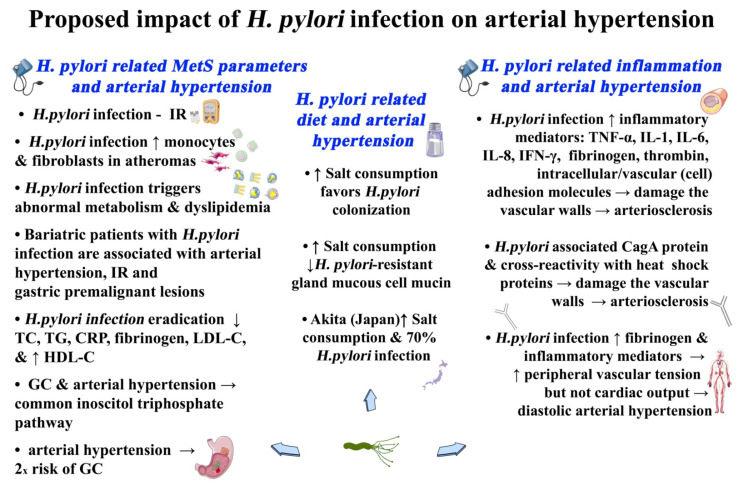
Proposed pathogenetic mechanisms, owing to *H. pylori* infection-related MetS, that may contribute to arterial hypertension. CagA, cytotoxin-associated gene A; CRP, C-reactive protein; HDL-C, high-density lipoprotein cholesterol; *H. pylori, Helicobacter pylori*; IL, interleukin; IR, insulin resistance; LDL-C, low-density lipoprotein-cholesterol; MetS, metabolic syndrome; TC, total cholesterol; TG, triglycerides; TNF, tumor necrosis factor.

The mechanisms involved in the pathophysiology of *H. pylori*-related arterial hypertension appear to be multifactorial, including diet, inflammatory processes, and MetS-related parameters (Figure 1).

### 2.1. H. pylori and Mets-Related Diet and Arterial Hypertension

High salt consumption can cause the development of arterial hypertension [65,66]. Focusing on a *H. pylori*-related diet, high-salt consumption, as a recognized risk factor for arterial hypertension, also favors *H. pylori* colonization [67,68,69]; high salt intake could stimulate the gastric mucosa and make it susceptible to *H. pylori* infection [68]. Indeed, experimental data in mice have revealed that high-salt consumption facilitates the development of *H. pylori* colonies [68]. Likewise, high-salt intake increases the surface mucous cell mucin with an affinity to *H. pylori*, reduces the *H. pylori*-resistant gland mucous cell mucin, and injures the gastric mucosal gel layer [69]. As additional evidence, the 1991 EUROGAST study, by investigating worldwide *H. pylori* infection rates, reported that the Akita area in Japan had the highest incidence of *H. pylori* infection (70%) [67]. Remarkably, Akita is located in the Tohoku region, where the diet is particularly high in salt compared to the other regions of Japan. The high *H. pylori* infection incidence in Akita, a developed region with raised standards of hygiene, indicates an association between *H. pylori* infection rates and high-salt diets [70]. Thus, the incidence of arterial hypertension in a region with a high-salt diet might be closely connected to *H. pylori* infection, though this may also depend on the degree of hygiene and sociocultural parameters [71,72].

**Figure 2:**
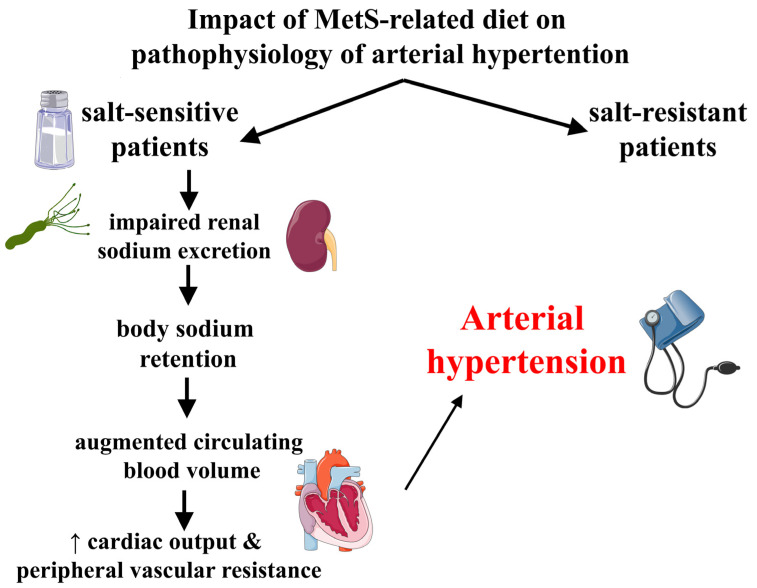
Simplified pathogenetic approach of diet (i.e., salt) impact on arterial hypertension. Patients with *Helicobacter pylori* infection belong also to the salt-sensitive category.

It is important to note that, in relation to salt in the diet, there are two forms of hypertensive patients: salt-sensitive, as in the case of *H. pylori* infection, in whom blood pressure rises with high salt intake; and salt-resistant, whose blood pressure does not increase with salt intake (Figure 2) [73,74,75]. Salt-sensitive patients display impaired sodium excretion through the kidneys, which results in body salt retention, augmented circulating blood volume, increased cardiac output, and eventually enhanced peripheral vascular resistance, leading to arterial hypertension [73,74,75,76,77]. In order to compensate the expanded intravascular volume, renal and peripheral vascular resistance are reduced, renal blood flow (RBF) rises, and renal sodium excretion increases in salt resistance. Nevertheless, the decline in peripheral vascular resistance with a high-salt diet is absent in salt-sensitive patients, a phenomenon that is connected with compromised vascular endothelial function and abnormally enhanced vasoconstrictor reactions in vascular smooth muscle cells [78,79]. Elements that affect renal sodium absorption, and therefore salt-sensitive arterial hypertension, include the aforementioned RAAS [80] angiotensin II [81] aldosterone, as well as the aforementioned SNS [82,83]. Angiotensin II induces sodium retention by increasing tubular sodium reabsorption [81] and reducing RBF [84]. Experimentally increased aldosterone, in aldosterone-treated animal models [85], and obesity [86] appear to stimulate sodium reabsorption from the epithelial sodium channel in the distal tubule through the activation of mineralocorticoid receptors during salt intake, leading to fluid retention and finally to salt-sensitive arterial hypertension. It is important to note that high salt consumption appears to be a risk factor for MetS; several lifestyle elements are closely related with MetS, including a high-salt diet [87,88,89].

### 2.2. H. pylori-Related MetS-Induced Inflammation and Arterial Hypertension

Inflammatory processes are an important feature of MetS components; obesity, dyslipidemia, IR, and atherosclerosis are all closely connected with inflammatory processes [90,91].

Focusing on *H. pylori*-related inflammatory processes, chronic inflammation due to *H. pylori* infection [46] activates a variety of mediators (Table 2) that have been linked to MetS-related endothelial cell dysfunction [19,92]. Indeed, *H. pylori* increases the levels of inflammatory mediators, such as MetS-related tumor necrosis factor (TNF)-α, interleukin (IL)-1, IL-6, IL-8, interferon (IFN)-γ, fibrinogen, thrombin, intercellular adhesion molecule, and vascular cell adhesion molecule; these MetS-related inflammatory mediators directly or indirectly damage the vascular walls, thereby triggering atherosclerosis [39,93,94,95,96]; *H. pylori*-mediated inflammation has been linked to atherosclerosis [97]; and the aforementioned inflammatory mediators have been involved in the pathophysiology of MetS-related arterial hypertension [98,99].

Specifically, CagA (cytotoxin-associated gene A) is a critical *H. pylori* virulence factor connected with a greater inflammatory response [100,101] and *H. pylori*-related CagA could especially be involved in the development of atherosclerosis [102]; by introducing polymerase chain reaction (PCR), *H. pylori* DNA has been detected in the atherosclerotic plaques of patients with severe coronary artery disease, and *H. pylori* infection accompanying the expression of CagA proteins is significantly connected with coronary artery disease. Coronary artery disease patients with CagA display more extensive damage of the coronary artery lumen and more frequently post-percutaneous transluminal coronary angioplasty (PTCA) with stent insertion re-stenosis of the coronary artery; and a *H. pylori* eradication regimen improves the reduction in the coronary artery lumen in these post-PTCA patients, possibly owing to the reduction of *H. pylori* pro-inflammatory cytokine release and the attenuation or elimination of *H. pylori*-induced chronic inflammatory prosses [102]. It appears that the antibodies against CagA can directly cross-react with the surface antigen of the blood vessel wall [103], induce lymphocyte proliferation, and provoke the host to release several pro-inflammatory agents, such as the aforementioned IL-1, TNF-α, IL-1, fibrinogen, and CRP [104,105]; as stated, such pro-inflammatory mediators are also related with MetS [106]. The subsequent inflammatory response increases the number of white blood cells and CRP concentrations in the circulation, triggers inflammation of the arterial wall, ultimately leading to vascular endothelial cell injury and dysfunction, smooth muscle cell proliferation, and atherosclerosis accompanied by arterial hypertension [107]. The existence of common epitopes, between the *H. pylori* CagA antigens and some peptides expressed by endothelial cells and smooth muscle cells is a phenomenon known as molecular mimicry. Therefore, in patients with a predisposition for arterial hypertension, the CagA antibodies could interfere with smooth-muscle cell function, thereby inducing arterial hypertension. However, this postulation remains to be elucidated. Of note, as in the case of CagA, *H. pylori*-induced vacuolating cytotoxin A (VacA) is also connected with a gastric inflammatory response, thereby contributing to gastric carcinogenesis. Moreover, VacA exhibits chemotactic activities in the bone marrow-derived mast cells and stimulates bone marrow-derived mast cells to induce proinflammatory cytokines that damage the blood–brain barrier [49]. Furthermore, VacA promotes intracellular survival of the bacterium and activated monocytes (possibly infected with *H. pylori* due to defective autophagy, resulting in *H. pylori* replication in autophagic vesicles) might access the brain owing to blood–brain barrier disruption (the Trojan horse theory), thereby contributing to *H. pylori*-related MetS brain pathologies [45].

More specifically, the mechanism linked with chronic inflammation—the antigen cross-reactivity of *H. pylori* with epitopes—triggers an autoimmune response leading to inflammatory vascular endothelial damage [108], which is a feature of MetS, predisposing to MetS-related ischemic disorders [19]. Essentially, an autoimmune response, which involves cross-reactivity between CagA antibodies and vascular wall antigens, signifies that these antibodies may contribute to the activation of inflammatory cells within atherosclerotic lesions. Moreover, antigenic cross-reactivity between human heat shock proteins and *H. pylori* has been connected with coronary artery calcification and atherosclerosis, accompanied by arterial hypertension [109,110].

Furthermore, atrophic gastritis owing to *H. pylori* infection with or without MetS induces vitamin B12 and folic acid deficiency, which results in hyperhomocysteinemia with subsequent damage to the vascular endothelial cells [111,112]; *H. pylori*-induced chronic gastritis can result in vitamin B12 and folate malabsorption, leading to methylation by 5- methyl-tetrahydrofolic acid failure and, hence, homocysteine accumulation. Hyperhomocysteinemia is a potent contributor to vascular disorders [111], an independent risk factor for MetS-related atherosclerosis [113], and a risk factor for arterial hypertension [114]. Specifically, homocysteine inhibits the secretion of nitric oxide by endothelial cells and triggers thromboxane-mediated platelet aggregation and vasoconstriction; it causes endothelial cell injury, promotes smooth muscle cell proliferation, and attenuates the protective effect of endothelial cell-derived relaxation agents [115]; and hyperhomocysteinemia, hyperfibrinogenemia, and high lipoprotein-a (a low density lipoprotein-like particle which contains the plasminogen homologue apo(a) disulfide-bound to apo B), as three ‘non-conventional’ coronary artery disease risk factors, may promote the occurrence of atherosclerosis and sequelae, including arterial hypertension [116].

It is important to note that diastolic blood pressure mostly depends on peripheral resistance, whereas systolic blood pressure predominantly depends on cardiac output. Patients with *H. pylori* infection display higher concentrations of MetS-related fibrinogen, an indicator of vascular inflammation that inhibits the production of nitric oxide and nitric oxide synthase (NOS) from the vascular endothelium, leading to vasoconstriction and augmented peripheral vascular tension [117]. The elevated concentrations of fibrinogen and inflammatory cytokines mentioned above in *H. pylori*-infected subjects result in high peripheral vascular tension but not cardiac output, which may explain, at least partly, why *H. pylori* infection was reported to be associated with diastolic blood pressure but not systolic blood pressure in some studies [118]. Of note, fibrinogen is a risk factor for hypertension, and its levels predict acute coronary and ischemic vascular events; moreover, they are higher in patients with future ischemic stroke development than those without [119,120,121]. Finally, *H. pylori* increases ammonia in the intestine and the accompanying intestinal spasms can impair absorption within the digestive tract, thus leading to enhanced reabsorption of sodium via the kidneys, causing arterial hypertension [63].

**Table 2:** Proposed pathogenetic mechanisms of *H. pylori* and MetS-induced inflammation on arterial hypertension.

Mechanism	Comment	References
Upregulation of inflammatory mediators	MetS-related inflammatory mediators damage directly or indirectly the vascular walls and trigger atherosclerosis	[93,94,95,96,97]
Cag A	*H. pylori* virulence factor connected with: (1) greater inflammatory response, (2) atherosclerosis, and (3) coronary artery disease.	[100,101,102]
VacA	*H. pylori* virulence factor connected with: (1) gastric inflammation and carcinogenesis, (2) chemotactic activation of bone marrow-derived mast cells and stimulation and damage to the blood–brain barrier, (3) promotion of intracellular *H. pylori* survival, and (4) brain access of activated monocytes (the Trojan horse theory)	[45,49]
Cross reactivity of *H. pylori*	Autoimmune response triggering by *H. pylori* cross-reactivity → vascular endothelial damage → MetS-related ischemic disorders	[108,109,110]
Atrophic gastritis	Vitamin B12 and folic acid deficiency induced by *H. pylori* and/or MetS → hyperhomocysteinemia resulting in: (1) vascular endothelial cells damage and (2) MetS-related atherosclerosis—arterial hypertension	[111,112,113,114,115,116]
Diastolic blood pressure	Higher concentrations of *H. pylori*/MetS-related fibrinogen → inhibition of endothelial nitric oxide and nitric oxide synthase → vasoconstriction and augmented peripheral vascular tension but not cardiac output → isolated diastolic blood pressure	[117,118]

CagA, Cytotoxin-associated gene A; *H. pylori, Helicobacter pylori*; MetS, metabolic syndrome; VacA, vacuolating cytotoxin A.

### 2.3. H. pylori-Related MetS Parameters and Arterial Hypertension

Regarding *H. pylori*-related MetS parameters, *H. pylori* infection is a potential contributor to IR, the major underlying mechanism responsible for MetS [46], which also plays an important role in the pathogenesis and progression of arterial hypertension-triggered target organ injury [122,123]. MetS contributes to an increased risk of developing atherosclerosis [124], and, in this respect, invasion of *H. pylori* leading to atheroma has been detected by introducing PCR [102,125]]. Likewise, direct *H. pylori* colonization in the arterial walls has been observed [126]. Moreover, *H. pylori* reacts with monocytes and stimulates fibroblast proliferation in atheroma [127]. Thus, *H. pylori* has been associated with MetS-related atherosclerosis via a variety of mechanisms, thereby potentially triggering arterial hypertension. *H. pylori* infection might independently be involved in atherosclerosis and arterial hypertension via mechanisms distinct from the conventional causes of atherosclerosis, such as the three non-conventional coronary artery disease risk factors—homocysteine, fibrinogen, and lipoprotein(a) [39,111,128].

MetS-related dyslipidemia is also linked with arterial hypertension [129], and, in this regard, chronic *H. pylori* infection can trigger abnormal lipid metabolism of the host including, besides lipoprotein(a) [128], low-density lipoprotein cholesterol (LDL-C), high-density lipoprotein cholesterol (HDL-C), and total cholesterol (TC) [130,131]. Likewise, *H. pylori* inflammatory cytokines involved in MetS-related arterial hypertension appear to alter lipid profiles. TNF-α inhibits the action of lipoprotein lipase, and transfers lipids from the adipose tissue, so that the concentrations of triglycerides (TG) in the circulation increase, whereas the concentrations of HDL-C reduce [132]. Moreover, IL-6 and TNF-α increase liver cholesterol production by affecting the expression of *3-hydroxy-3-methyl glutaryl coenzyme A reductase* gene, and inhibit *cholesterol hydroxylase* to decrease liver cholesterol catabolism [133]. In contrast, *H. pylori* eradication significantly decreases the concentrations of TC, TG, CRP, fibrinogen, and LDL-C, whereas it increases the concentrations of HDL-C [134,135]. Therefore, eradication of *H. pylori* infection reduces the occurrence of dyslipidemia, thereby potentially preventing the occurrence of MetS-related cardiovascular disease accompanied by arterial hypertension. Beyond dyslipidemia and arterial hypertension, *H. pylori* eradication also ameliorates other MetS-related parameters, such as body mass index (BMI) [136], IR [137], total oxidant status [138,139], and fibrinogen, an independent risk factor for cardiovascular disease [39]. Focusing on BMI, some other investigators reported that *H. pylori* eradication seems to restore ghrelin by increasing gastric ghrelin secretion, leading to increased plasma ghrelin levels, increased appetite and a rise in BMI, though a causative relationship between *H. pylori*-connected serum ghrelin decline and food intake and obesity has not been established. Since other data indicate that plasma ghrelin levels are lowered by following a *H. pylori* eradication regimen, further research is needed to clarify this issue.

Interestingly, recent data indicate that MetS-related sarcopenia, *H. pylori* infection, dyslipidemia, arterial hypertension, diabetes mellitus, smoking, alcohol consumption, and diet (salty and/or spicy diets) are associated with precancerous gastric mucosa lesions, including gastric atrophy, intestinal metaplasia, and dysplasia [140]. In this regard, MetS is associated with malignances, including *H. pylori*-related upper and lower gastrointestinal tract cancers, and besides other MetS-related parameters, arterial hypertension could be a key parameter [46,141,142]. Biochemical reactions induced by dysregulated MetS parameters, including arterial hypertension, affect the host’s general condition and organ-specific microenvironment, leading to increased cancer recurrence and mortality [141]. Patients with arterial hypertension show a two-fold increased risk for gastric cancer (GC) development. Both arterial hypertension and GC may share a biochemical pathway of elevated inositol triphosphate and cytosolic calcium that could contribute to the pathogenesis of arterial hypertension and gastric carcinogenesis. Moreover, arterial hypertension is frequent in survivors of malignancies, and management of preexisting or incident arterial hypertension in these patients is crucial to reducing the risk of heart failure and other cardiovascular diseases [143]. Remarkably, recent data indicate that bariatric patients with *H. pylori* infection display significantly higher than baseline rates of the mentioned gastric pre-malignant lesions, including gastric atrophy and intestinal metaplasia accompanied with IR and arterial hypertension [144].

In line with the latter results, other investigators reported that bariatric patients also exhibit baseline occurrence of MetS-related parameters, such as arterial hypertension, dyslipidemia, IR, and even significant cyclooxygenase-2 (COX-2) induction. In contrast, bariatric surgery has been shown to decrease the magnitude of MetS-related comorbidities, such as arterial hypertension and dyslipidemia, and even remission of type 2 diabetes mellitus by normalizing peripheral insulin sensitivity and increasing pancreatic beta-cell sensitivity to glucose [145].

In this context, COX-2 also appears to be implicated in MetS components, including hypertension-related carcinogenesis [145]. It is mutagenic and tumorigenic in vitro, is frequently overexpressed in multiple malignant cells, and is associated with increased invasiveness and poor prognosis of tumors, including *H. pylori*-related upper and lower gastrointestinal tract malignancies [46,146]. *H. pylori*-induced COX-2 overexpression enhances prostaglandin synthesis, which has been proved to promote the development, proliferation, and metastasis of cancer cells [146]. On the other hand, although COX-2 selective inhibitors attenuate inflammation and suppress oncogenesis, their clinical use is connected with potential side effects, most remarkably those within the cardio-cerebrovascular system, including arterial hypertension, myocardial infarction, and stroke [147]. Specifically, *H. pylori* induces an immune response via COX-2 stimulation, which increases the production of prostaglandin and nitric oxide [112], and the *H. pylori* cell wall lipopolysaccharide (LPS) triggers Toll-like receptor-4, which stimulates secondary mediators, including mitogen-activated protein kinase, extracellular-signal-regulated kinase, c-Jun N-terminal kinase, and c-p38 kinase, and further stimulates NOS and COX-2 gene expression [148,149]. This immune reaction to LPS might increase the risk of atherosclerosis [150]. COX-2 has been connected with pro-inflammatory/pro-atherogenic mechanisms, owing to its overexpression in monocyte-derived macrophages, existing in the atherosclerotic lesions [151]. Likewise, COX-2 expression may play a role in the progression of atherosclerosis and in the induction of plaque rupture [152]. Myocardial infarction and stroke are direct outcomes of atherosclerotic plaque rupture induced by prostaglandin-E2-dependent matrix-degrading metalloproteinases [153].

Regarding MetS-related arterial hypertension, combined arterial hypertension and atherosclerotic plaques might raise the risk of cardio-cerebrovascular severe events and mortality [154]. COX-2 is the principal source of intravascular reactive oxygen species creation, and, in arterial hypertension and diabetes, this seems to be the result of an interaction between COX-2-induced prostaglandins, lower activity of oxidase, RAAS, nicotinamide adenine dinucleotide phosphate, and bone morphogenic protein 4, as a concentrated pathophysiological cascade in stimulating and preserving endothelial dysfunction [155,156]. Moreover, in patients with arterial hypertension, alterations in IL-12, IL-23, IL-27, IL-35, and IL-37 concentrations are connected with the development of carotid atherosclerotic plaque [157,158]. All aforementioned cytokines are also associated with *H. pylori* infection [159,160,161,162].

### 2.4. H. pylori and MetS-Related NAFLD and Arterial Hypertension

NAFLD, as the hepatic component of MetS, is associated with *H. pylori* infection, which appears to contribute to its development and progression [53]. Recent data indicate that *H. pylori* infection is connected with IR and augmented intestinal permeability, which could contribute to the development of NAFLD [163]; and active *H. pylori* infection is independently positively associated with the severity of nonalcoholic steatohepatitis (NASH) and fibrosis, findings suggesting probable clinical implications [53]. In this regard, among patients with NAFLD, the prevalence of arterial hypertension varies from 40–70% and relative studies have shown that NAFLD is strongly related to augmented risk of incident arterial prehypertension and hypertension [164,165]. Likewise, in the last few years, numerous cross-sectional studies have shown that the occurrence and severity of NAFLD are connected with augmented blood pressure and the occurrence of arterial prehypertension and hypertension [166,167]. Moreover, two- to three-fold rise in the incidence of arterial hypertension was confirmed in relative prospective epidemiological reports in France and Germany, after observational periods of nine and five years, respectively [168,169]. In addition, relevant data from Finland indicated that, among the hypertensive or normotensive participants, 24-h daytime and night-time systolic or diastolic blood pressure were significantly higher among participants with hepatic steatosis than without (estimated by ultrasound), while this association at a non-dipping status was marginally non-significant [170]. Ultrasonography revealed fatty liver more often when non-dipping or reverse dipping was found in 24-h ambulatory blood pressure monitoring in a group of hypertensive patients, and baroreceptor sensitivity was decreased with augmented blood pressure variability among patients with NAFLD [171,172].

Cytokeratin (CK)-18 is a well-known marker of hepatocellular-specific apoptosis with its additional potential value as a noninvasive indicator in predicting the severity of inflammation, steatosis, and fibrosis [173]. CK-18 is not normally found in vascular smooth muscle, but is highly expressed during the development of atherosclerotic plaques and has been shown to be elevated in hypertensive patients with NAFLD [174]. Indications are available displaying correlations of CK-18 with blood pressure, thus implying its crucial role in the pathophysiology of such disorders [174,175]. Specifically, CK-18 is an intermediate filament, the cleavage of which is an early event during apoptosis following activation of effector caspases and its immunoreactivity is significantly higher in the foveolar epithelium of *H. pylori*-positive gastritis compared with both *H. pylori*-negative gastritis and controls; higher expression of CK-18 in the foveolar epithelium is noticed in patients with CagA positive *H. pylori*-induced gastritis [176]. Furthermore, associations between liver enzymes, including serum aspartate aminotransferase, alanine aminotransferase, and gamma-glutamyl transferase and hypertension are well established. Likewise, several studies have reported positive associations between these enzymes and arterial hypertension in NAFLD [177]. Apparently, NASH associated with active *H. pylori* infection is characterized by increased levels of the aforementioned liver function tests [53].

The following proposed mechanisms (Table 3) appear to be involved in the pathophysiology of *H. pylori* and MetS-related NAFLD on arterial hypertension:

**Hypertension** **1.** *H. pylori and MetS-related NAFLD may promote arterial hypertension by inducing systemic inflammatory processes.*

The liver, as an immunological organ with a distinctive tissue structure and cellular composition, comprising abundant Kupffer cells (residential macrophages) and immune cells, can provoke secretion of cytokines to defend against invading microorganisms and environmental challenges and release them into systemic circulation, thereby inducing systemic inflammation [178]. Correspondingly, recent clinical data showed that patients with NAFLD had significantly compromised cardiac and autonomic function, and, in particular, the raised levels of TNF-α and CK-18 (as markers for liver injury) in NAFLD patients were independently connected with augmented sympathetic activity and reduced parasympathetic activity [179]. Moreover, NAFLD has been reported to be related to systemic inflammatory responses, characterized by elevated levels of cytokines IL-6 and TNF-α [180,181]. Proinflammatory cytokine, such as the mentioned *H. pylori*-related TNF-α and IL-6, have been revealed to regulate the expression of the mentioned RAAS components, especially angiotensinogen production in the liver and kidneys, further promoting systemic and local angiotensin II formation and angiotensin II-dependent hypertension [182]. It appears that under systemic inflammation induced by NAFLD, local inflammatory infiltration in the vasculature, kidney, and adipose tissue might accelerate the development and progression of arterial hypertension, and thus further research is needed.

**Hypertension** **2.** *H. pylori and MetS-related NAFLD may promote arterial hypertension by inducing augmented oxidative stress.*

NAFLD has been suggested to be highly associated with oxidative stress [183]. In this respect, reactive oxygen species may play a role in *H. pylori*-related MetS extragastric systemic disorders, including atherosclerosis involved in arterial hypertension [184].

**Hypertension** **3.** *H. pylori and MetS-related NAFLD may promote arterial hypertension by inducing IR.*

Accumulating experimental and clinical data have highlighted a close and causal relationship between NAFLD and *H. pylori*-related IR, a key component of MetS [53,185,186]. Mechanistically, in the setting of IR, excess free fatty acids released by adipose tissue stimulate ectopic fat deposits, including perivascular fat and renal sinus fat, thereby increasing the risk of arterial hypertension [187].

**Hypertension** **4.** *H. pylori and MetS-related NAFLD gastrointestinal dysbiosis may promote arterial hypertension*.

*H. pylori*-related gastrointestinal dysbiosis has been associated with MetS-related systemic disorders, including arterial hypertension and NAFLD [45]. Currently, it is well recognized that NAFLD and gastrointestinal dysbiosis have been connected, and dysbiosis has been associated with the development and possibly the progression of NAFLD [188]. Strikingly, in the sequencing analysis of fecal microbiota, NAFLD patients have exhibited decreased richness and diversity of gut microbiota, especially increased *Bacteroides* and decreased *Prevotella*, which are closely connected with the production of short-chain fatty acids [189]; these changes could further contribute to arterial hypertension. In parallel with gastrointestinal dysbiosis, the investigation of fecal microbiome characteristics in NAFLD patients implied that NAFLD was linked with enriched genes encoding proteins essential in the biosynthesis of lipopolysaccharide, which can disrupt the intestinal barrier [189]. Subsequently, increased translocation of bacteria, including *H. pylori* [190], microbial-associated molecular patterns and gastrointestinal metabolites, elicit intestinal and hepatic inflammatory responses, thereby accelerating the progression of NAFLD. Moreover, systemic lipopolysaccharide levels are significantly raised in patients with NASH [191] and may stimulate systemic inflammation and induce arterial hypertension.

**Hypertension** **5.** *H. pylori and MetS-related NAFLD may promote arterial hypertension by increasing vasoconstriction and decreasing vasodilation.*

The liver is the central site of the elimination of asymmetrical dimethylarginine (ADMA), which is an endogenous inhibitor of the mentioned NOS. There is evidence that ADMA is connected with endothelial dysfunction and vascular flow alterations in patients with arterial hypertension. Notably, circulating ADMA is positively related with the prevalence of MetS [192]. Moreover, clinical studies have revealed that NAFLD patients exhibit augmented levels of circulating ADMA, even independent of traditional cardiovascular risk factors [193]. Furthermore, NAFLD patients exhibit impaired eNOS (endothelial NOS) function in systemic circulating platelets [194], which may further lead to reduced nitric oxide production and impair nitric oxide-dependent vasodilatation. Additionally, RAAS activation by systemic inflammation and augmented production and secretion of RAAS components, including angiotensin II, can also increase vasoconstriction. Prospective studies also suggest that NAFLD is an independent pathogenic factor in the development and progression of arterial stiffness, which can, in turn, modulate vessel contractile function [195]. Therefore, it is possible that NAFLD can directly increase vasoconstriction and drive the development of arterial hypertension, and thus further investigation is needed.

**Hypertension** **6.** *H. pylori-related MetS-NAFLD genetic and epigenetic modifications may promote arterial hypertension.*

Generated gene–metabolite–disease interaction networks show that NAFLD and arterial hypertension are interlinked by molecular signatures. Although a large amount of evidence exists for the relationship between NAFLD and arterial hypertension, there is little evidence on the genetic association between NAFLD and arterial hypertension. Bioinformatic analysis revealed that the *AGTR1* (angiotensin receptor type 1) gene, also connected with *H. pylori* infection and MetS [196,197], might be involved in several signaling pathways connected with the development of NAFLD [198]. In a recent prospective cohort study, the gain-of-function A1166C (rs5186) variant in the *AGTR1* gene represented a strong predictor of incident NAFLD and related arterial hypertension [199]. Thus, despite the previous unsuccessful clinical trial of angiotensin receptor blockers in managing fibrosis in NAFLD patients [200], patients with the *AGTR1 A1166C* variant may exhibit a subtype of NAFLD that may benefit more from the personalized usage of angiotensin receptor blockers.

## 3. Concluding Remarks

*H. pylori* infection displays a pleiotropic effect beyond the alimentary tract and mounting evidence associates the infection with MetS, including arterial hypertension. The above-described mechanistic proposals have presented a pathogenetic substrate, showing how *H. pylori* infection may possibly exert its action and influence MetS-related arterial hypertension. Further research is warranted, however, to elucidate in depth the potential impact of *Helicobacter pylori*-related MetS on arterial hypertension, a serious public health problem with high global incidence and prevalence that continues to increase and may contribute to globally high levels of morbidity and mortality [1].

Identifying *H. pylori* and MetS-related NAFLD as an important risk factor for hypertension may be helpful for improving the risk prediction, identifying primary preventive strategies, and selecting a therapeutic program for arterial hypertension and associated systemic MetS-related pathologies, including cardiovascular disorders.

## Figures and Tables

**Table 1:** Synopsis of potential etiologic agents of MetS-related arterial hypertension.

Category	Comment	References
Genetic factors	*ATP2B1* gene polymorphisms *rs2681472* and *rs17249754*	[12]
Diet	High Na^+^/K^+^ intake and western-type of diet	[13,14,15]
Adiposity	Activation of the SNS and RAAS, and sodium retention	[16]
Smoking	Mainly via stimulation of the SNS	[18]
Endothelial dysfunction linked with MetS	Excessive release of vasoconstrictive agents and defective secretion of smooth-muscle relaxing mediators	[17]
Gut microbiota dysbiosis	Via production, modification, and degradation of microbial-derived bioactive metabolites	[15,21,22,23]
Inflammatory mechanisms	Overstimulated immune system induction of pro -inflammatory cytokines and chemokines overexpression, cell infiltration and oxidative stress	[24,25]
NAFLD linked with MetS	Mainly but not exclusively with hyperinsulinemia—insulin resistance	[27]
MetS-related brain neurodegenerative disorders	Disruption of blood–brain barrier, triggering neuroinflammation and amyloid disorders and decreasing the function of the cerebral blood vessels, including reduced cerebral blood flow, altered brain autoregulation, and compromised neurovascular coupling	[28,29,30,31]
MetS–related cancer development	Arterial hypertension displays a two-fold increased risk for GC development	[32,33]

MetS, metabolic syndrome; NAFLD, nonalcoholic fatty liver disease; GC, gastric cancer; RAS, renin-angiotensin system; SNS, sympathetic nervous system.

**Table 3:** Promotion of arterial hypertension by proposed pathogenetic mechanisms of *H. pylori* and MetS-related NAFLD.

Mechanisms
**Promotion of arterial hypertension by:**
• Augmented oxidative stress
• Increased insulin resistance
• Gastrointestinal dysbiosis • Increased vasoconstriction and decreased vasodilation
• Genetic and epigenetic modifications

*H. pylori*, *Helicobacter pylori*; MetS, metabolic syndrome; NAFLD, nonalcoholic fatty liver disease.
